# Retraction: Facile, one-pot biosynthesis and characterization of iron, copper and silver nanoparticles using *Syzygium cumini* leaf extract: As an effective antimicrobial and aflatoxin B_1_ adsorption agents

**DOI:** 10.1371/journal.pone.0329780

**Published:** 2025-08-06

**Authors:** The PLOS One Editors

After this article [1] was published, concerns were raised about Figs 1-2.

Specifically, when aspect ratio is altered, the following panels appear to partially or fully overlap:

- Fig 1C in [1], Fig 3A of [2] (retracted in [3]), and Fig 3f of [4] (retracted in [5]).
- Fig 2A in [1] and Fig 2d of [4] (retracted in [5]).
- Fig 2B in [1], Fig 2b and Fig 2f of [4] (retracted in [5]).
- Fig 2C in [1], Fig 2c and Fig 2e of [4] (retracted in [5]).

The corresponding author provided underlying image data for Fig 1 and a revised figure with an updated panel for Fig 1C, stated to be from a parallel experiment from the time of the original experiments. The corresponding author notified the journal of the panel overlap in Fig 2C and provided replacement images for all panels in this figure. However, these images raised further integrity concerns and the above concerns remain unresolved.

In light of the above concerns, which call into question the reliability and integrity of the article’s results and conclusions, the *PLOS One* Editors retract this article.

MAsifA, EZ, and MArifA did not agree with the retraction. JI and AAR either did not respond directly or could not be reached.

Figs 1C and 2A-C report material from [2,4], published in 2020 by Elsevier. The reused content was modified and not offered under a CC BY license and is therefore excluded from this article’s [1] license.
